# Do Alopecia Areata and Hair Colour Have a Shared Genetic Component?

**DOI:** 10.1111/exd.70299

**Published:** 2026-06-17

**Authors:** Leonie Rieger‐Molitor, Carlo Maj, Silke Redler, Yasmina Gossmann, Stephan Ripke, Lynn Pethukova, Angela M. Christiano, Markus M. Nöthen, F. Buket Basmanav, Regina C. Betz

**Affiliations:** ^1^ Institute of Human Genetics University of Bonn, Medical Faculty & University Hospital Bonn Bonn Germany; ^2^ Institute for Genomic Statistics and Bioinformatics University of Bonn, Medical Faculty & University Hospital Bonn Bonn Germany; ^3^ Department of Psychiatry and Psychotherapy Charité – Universitätsmedizin Berlin Germany; ^4^ Ronald O. Perlemen Department of Dermatology NYU Langone Health New York New York USA; ^5^ Department of Population Health NYU Langone Health New York New York USA; ^6^ Department of Genetics and Development Columbia University New York New York USA

**Keywords:** alopecia, genetics, hair colour, immunology

## Abstract

Alopecia areata (AA) is a common T‐cell mediated autoimmune disorder, which leads to sudden, non‐scarring hair loss. An association between hair pigmentation and AA pathogenesis has long been postulated. Recent epidemiological evidence supports this link, indicating that individuals with darker hair colours have a higher risk of AA, while those with lighter hair colours exhibit a lower risk. This study aimed to investigate whether a shared genetic basis between hair colour determination and AA risk underlies this observation. To explore this, we analysed data from our AA genome‐wide association study (GWAS) of 16 310 Caucasians from the US and Central Europe and a recently published GWAS of hair colour that involved 343 234 UK Biobank participants. Genetic correlations between the two traits were investigated using both polygenic risk score and linkage disequilibrium score regression (LDSC) analyses. No evidence was found for a strong, broadly shared genetic component for hair colour and AA. However, a suggestive weak inverse association with genetically predicted blond hair and AA risk was observed. Given the modest sample size of the AA‐GWAS (~4100 cases), these findings are likely underpowered and should be interpreted as exploratory and hypothesis‐generating, warranting replication in larger, independent cohorts.

## Graphical abstract

The aim of our study was to determine whether hair colour and the risk of developing alopecia areata (AA) share a genetic basis. While we found no evidence for a broadly shared polygenic background, our analyses revealed suggestive evidence that genetically predicted blond hair is associated with a reduced risk of AA.

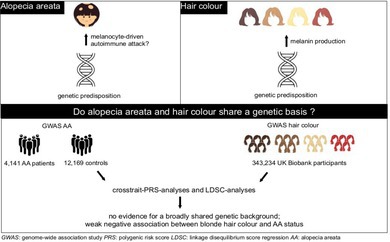

## 1 Background

Alopecia areata (AA) is a common hair loss disorder that affects both sexes and has a lifetime prevalence of around 2% [1]. Hair loss usually commences in the form of patches (patchy AA) and may progress to complete baldness of the scalp (alopecia totalis) or a total loss of body hair (alopecia universalis). AA has an unpredictable and variable disease course, which may include periods of remission and relapse or persistent and extensive hair loss. The sudden onset and unpredictable course of AA have major adverse psychological consequences for those affected.

From a pathological perspective, AA is a T‐cell mediated autoimmune disorder targeting the hair follicle (HF), with the immunological attack primarily directed at the bulb of the anagen HF. Notably, this process occurs in temporal and spatial overlap with hair melanogenesis, which is restricted to the anagen phase of the hair cycle. During this phase, melanocytes in the upper matrix of the hair bulb transfer melanosomes to the keratinocytes of the hair shaft. Previous authors have speculated that a melanocyte/melanogenesis‐driven autoimmune attack may represent the primary pathogenic mechanism in a subgroup of AA patients [2, 3, 4, 5, 6, 7]. Clinical observations, on the other hand, further suggest a relationship between hair pigmentation and AA manifestation. It is frequently reported in AA patients who have both pigmented and white/grey hair, that is unpigmented, that AA preferentially attacks the former and (relatively) spares the latter [6, 8, 9, 10, 11, 12]. Furthermore, research in AA patients suggests that regrowing hair is initially white and becomes repigmented over time [10, 13], although cases of persistent white hair regrowth have been documented [14, 15]. Several studies have also reported a higher risk of AA in dark‐haired individuals, and a lower risk for individuals with lighter hair colours [12, 16, 17]. However, as these findings are derived from observational data, potential confounding by ancestry and related pigmentation traits should be considered.

Taken together, these observations point towards a potential relationship between hair pigmentation and AA susceptibility and raise the question of whether shared genetic influences contribute to both hair colour determination and AA risk. Identifying genetic variants associated with both hair colour and AA may help to inform future studies aimed at elucidating the underlying biological pathways and their potential therapeutic implications.

Consistent with this, data from our genome‐wide association study (GWAS) of AA [18, 19], along with findings from other studies [20, 21], suggest some locus‐specific overlap between genetic variants associated with AA susceptibility and pigmentation traits. In detail, variants at syntaxin 17 (*STX17*), an autophagy‐related gene, are both significantly associated with the risk to develop AA and hair colour in humans and horses [18, 19, 20, 21]. Furthermore, *PMEL* located at a second AA GWAS locus 12q13.2 encodes for the melanocyte‐specific premelanosome protein that plays an essential role in the structural organization of premelanosomes and pigmentation, and genetic variants at this locus are also significantly associated with protein levels of PMEL [22].

## 2 Questions Addressed

Is there a broad overlap between genetic factors involved in the determination of hair colour and the risk for AA development?

## 3 Experimental Design

In order to explore this, we analysed genetic data (i.e., summary statistics) from both our own AA case–control GWAS cohort [18, 23] with additional 888 cases and 4626 controls (in total: 4141 AA patients and 12 169 healthy controls of Caucasian origin) and a recently published GWAS of hair colour derived from the data of 343 234 UK Biobank participants, who had been stratified according to hair colour [24]. The study was performed in accordance with the principles of the Declaration of Helsinki. All individuals provided written informed consent prior to the provision of blood samples for genotyping.

For the investigations, we applied both cross‐trait polygenic risk score (PRS) and linkage disequilibrium score regression (LDSC) analyses [25, 26, 27]. The PRS method estimates the lifetime risk of a given individual for a specific trait/disorder on the basis of multiple genetic markers [27]. A cross‐trait PRS approach investigates whether the PRS for one trait/disorder is associated with the phenotypic distribution in another trait (e.g., case vs. control status), thereby indicating a genetic overlap between the two traits/disorders. The present study investigated whether the PRS for blond, brown, or red hair was associated with AA by comparing the respective hair colour PRS in AA cases versus controls. LDSC is another statistical method that can be employed to compute the genetic correlation between complex polygenic traits from GWAS summary statistics. Here, LDSC was used to assess the shared genetic component of hair colour and AA.

PRS analysis was performed using clumping plus a thresholding approach and the PRSice tool v2 [26]. Clumping was conducted by considering 250 kb regions and an R2 of 0.1, and testing different *p*‐value thresholds for variant selection (i.e., 5.0E‐08, 1.2E‐06, 1E‐04, 1E‐03, 1E‐02, 5E‐02, 0.1, 0.2, 0.5, 1). To avoid batch effects, PRS associations were computed independently in five batches of samples, each of which had been genotyped separately, and regression analyses were performed including sex and PC1–PC10 as covariates. The single‐cohort PRS regression coefficients (beta and standard error) were then combined in a meta‐analysis using the restricted maximum‐likelihood (REML) estimator, as implemented in the R package metaphor. The genetic correlation between AA and each of the three hair colours was computed using the LDSC analysis and by considering the European linkage disequilibrium reference from the 1000 Genome Project.

## 4 Results

No significant associations were found between AA case status and the PRS for either brown or red hair colour (Bonferroni‐adjusted *p*‐value of best PRS = 0.32 and 0.84, respectively). On the other hand, our results revealed a negative association between the PRS for blond hair and AA case status (Bonferroni‐adjusted *p*‐value of best PRS = 0.017, z‐score −3.45), which aligns with previous evidence suggesting that light hair colour may confer a protective effect against AA risk.

The LDSC analysis yielded no statistically significant genetic correlations (gc) between AA case–control status and any of the three hair colours. However, the observed effect sizes suggested the existence of a positive correlation between AA case status and brown hair colour (gc = 0.11 SE = 0.08, *p* = 0.19), as well as negative correlations between AA case status and the two lighter hair colours (blond: gc = −0.29 SE = 0.053, *p* = 0.58; red: gc = −0.030 SE = 0.079, *p* = 0.70). The latter observation was consistent with the observed negative association between blond hair PRS and AA case status.

Of note, in the GWAS of hair colour, ~73% of the heritability for red hair was explained by the single locus *MC1R*. We therefore tested for an association between the leading variant in this locus (rs34357723; P__hair color_ = E‐308), and AA case–control status in our own AA cohort. This yielded no evidence for an association between the *MC1R* locus and AA (P = ~0.17).

## 5 Conclusions and Perspectives

Although our study generated no evidence for a broadly shared polygenic background for hair colour and AA, the finding of suggestive evidence for a negative association between genetically predicted blond hair and AA risk could be considered to be consistent with previous clinical observations indicating relative sparing of non‐pigmented hair in AA. Nevertheless, this weak association must be interpreted with caution and requires verification in larger cohorts. In this context, it should also be noted that PRS and LDSC capture related but distinct aspects of shared genetic architecture: whereas LDSC estimates genome‐wide genetic correlation based on the overall concordance of effect sizes across the genome, PRS evaluates whether aggregated allelic effects derived from one trait show predictive value in an independent target sample. Thus, a weak PRS association may be observed even when genome‐wide genetic correlation is not detected.

On the other hand, in the current study we examined genetic correlations between AA and hair colour by considering the genetic signals of common genetic variants, which are the usual focus of GWAS. However, common variants tend to have small effects and thus, diseases and traits with well‐resolved genetic architectures involving common variants have been studied in cohorts with tens of thousands of cases, which are far larger than the currently available AA GWAS cohorts. Given that the direction of effects we observed hereby were consistent with anecdotal and epidemiological evidence despite the relatively small AA GWAS cohort we employed, it is also likely that our study was underpowered to detect variants contributing to both phenotypes. This should also be clarified by future studies conducted in larger cohorts.

Another important consideration when interpreting the present results is that the GWAS of hair colour was performed in UKBiobank participants of British ancestry, while the AA case–control cohorts comprised Caucasians recruited in the US and Central Europe. To date, no large GWAS of AA or hair colour have been performed in other populations. Accordingly, when available, assessment of data from individuals of other genetic ancestries (e.g., African, east Asian, Middle Eastern, Northern European) may provide further insights into the presence of a shared genetic component between hair colour and AA.

Finally, rare or structural variants that are not captured by GWAS may also be involved in hair pigmentation and hair colour determination as well as the risk for AA development. A possible contribution by these should also be considered once more comprehensive data (e.g., whole genome sequencing and long‐read sequencing) become available.

In summary, using a genetics‐based approach, we investigated a potential overlapping etiological component between hair colour and AA risk. Our results provide limited evidence for a shared genetic component between hair colour and AA susceptibility, with a potential modest protective effect of genetically predicted blond hair. Importantly, as a genetics‐based association study, our analyses do not allow conclusions regarding specific biological or antigenic mechanisms, nor do they provide a basis for individual risk prediction for developing AA. Further studies are required to replicate these findings and to investigate the underlying biological pathways in more detail.

## Author Contributions

Data curation: Carlo Maj, Yasmina Gossmann, Stephan Ripke; Formal analysis: Carlo Maj; Funding acquisition: Lynn Pethukova, Regina C. Betz; Investigation: Leonie Rieger‐Molitor, Carlo Maj; Methodology: Carlo Maj, F. Buket Basmanav; Project administration: Regina C. Betz; Resources: Yasmina Gossmann, Silke Redler, Angela M. Christiano, Lynn Pethukova; Supervision: Carlo Maj, F. Buket Basmanav, Markus M. Nöthen, Regina C. Betz; Writing – original draft: Leonie Rieger‐Molitor, Carlo Maj; Writing – review and editing: Lynn Pethukova, Carlo Maj, Markus M. Nöthen, Stephan Ripke, F. Buket Basmanav, Regina C. Betz.

## Funding

This work was supported by Deutsche Forschungsgemeinschaft, EXC2151‐390873048; National Institutes of Health, K01AR075111.

## Conflicts of Interest

R.C.B. and A.M.C. received personal fees from Pfizer for activities unrelated to the submitted work. The other authors have no conflicts of interest to declare.

## Data Availability

The data that support the findings of this study are available on request from the corresponding author. The data are not publicly available due to privacy or ethical restrictions.
